# A siRNA mediated hepatic *dpp4* knockdown affects lipid, but not glucose metabolism in diabetic mice

**DOI:** 10.1371/journal.pone.0225835

**Published:** 2019-12-03

**Authors:** Sven Wolfgang Görgens, Kerstin Jahn-Hofmann, Dinesh Bangari, Sheila Cummings, Christiane Metz-Weidmann, Uwe Schwahn, Paulus Wohlfart, Matthias Schäfer, Maximilian Bielohuby

**Affiliations:** 1 Sanofi-Aventis Deutschland GmbH, Industriepark Hoechst, Frankfurt am Main, Germany; 2 Sanofi, Global Discovery Pathology, Translational In-vivo Models Framingham, MA, United States of America; Universidade do Estado do Rio de Janeiro, BRAZIL

## Abstract

Systemic inhibition of dipeptidyl peptidase 4 (*dpp4*) represents an effective and established treatment option for type 2 diabetes (T2D). The current study investigated in mice if a liver selective knock-down of *dpp4* by therapeutic siRNAs could be a novel, similarly effective treatment option for T2D. Furthermore, the potential effects on hepatic steatosis, inflammation and lipid metabolism were investigated after hepato-selective knock-down of *dpp4*. The knock-down efficiency and IC_50_ values of siRNAs targeting *dpp4* were analyzed in PC3 cells. In two independent studies, either db/db mice or C57BL/6J mice were injected intravenously with a liposomal formulation of siRNAs targeting either *dpp4* or a non-targeting control, followed by metabolically characterization. In comparator groups, additional cohorts of mice were treated with an oral *dpp4* inhibitor. In both animal studies, we observed a robust knock-down (~75%) of hepatic *dpp4* with a potent siRNA. Hepatic *dpp4* knockdown did not significantly affect glucose metabolism or circulating incretin concentrations in both animal studies. However, in obese and diabetic db/db mice hepatic steatosis was reduced and hepatic mRNA expression of *acaca*, s*cd1*, *fasn* and *pparg* was significantly lower after siRNA treatment. Systemic inhibition of the enzymatic *dpp4* activity by an oral dpp4 inhibitor significantly improved glucose handling in db/db mice but did not affect hepatic endpoints. These data demonstrate that a targeted reduction of *dpp4* expression in the liver may not be sufficient to improve whole-body glucose metabolism in obese and diabetic mice but may improve hepatic lipid metabolism.

## Introduction

Dipeptidyl-peptidase 4 (*Dpp4*) is a glycoprotein and exopeptidase of 110 kDa, which is ubiquitously expressed on the surface of many cells [1]. Dpp4 selectively cleaves N-terminal dipeptides from a variety of substrates, including the incretin hormones, namely glucagon-like peptide-1 (GLP-1) and glucose-dependent insulinotropic polypeptide (GIP) [2, 3]. The systemic inhibition of DPP4 by marketed drugs named gliptins, is being successfully used for reduction of hyperglycemia in type 2 diabetes (T2D) patients.

Global knockout of *dpp4* in mice largely mirrors treatment of humans with gliptins. Such knockout mice display improved glucose tolerance and increased GLP-1 plasma concentrations compared to wildtype controls and higher insulin sensitivity in diet-induced obesity (DIO) [4–6]. Dpp4 has been described as an adipokine, expressed and produced by adipocytes. In line with this observation of plasma dpp4 concentrations are also higher in obese compared to lean conditions [7, 8]. In addition to adipocytes, several other cell types express and release Dpp4 [2]. In mice, recent data demonstrate a high expression of *dpp4* in liver, which contributes significantly to the concentration of circulating dpp4 concentrations [9–11].

In subjects with ectopic liver fat accumulation, hepatic *dpp4* expression is elevated and positively correlates with markers of metabolic disorders such body weight, fasting blood glucose or HOMA-IR [11]. Interestingly, silencing of hepatocyte *dpp4*, but not the treatment with the systemic *dpp4* inhibitor sitagliptin, reduced visceral adipose tissue inflammation and improved glucose tolerance as well as insulin sensitivity in obese mice [12]. Therefore, it has been concluded that mainly hepatocyte-derived dpp4 promotes inflammation and insulin resistance in obesity, potentially also through auto- and paracrine mechanisms [10]. Most of these studies were performed in obese mice which were either normoglycemic or modestly hyperglycemic.

The present report specifies the use of small interfering RNAs (siRNA) as an efficient method to silence *dpp4* specifically in the liver of obese and severely hyperglycemic db/db mice. On the basis of initial optimization using a cell culture assay, finally a highly potent and stable siRNA could be identified. In combination with a specific liposomal formulation [13] hepatic *dpp4* knockdown in healthy C57BL/6 and insulin resistant db/db mice could be achieved, similar to recent work on a different target approach [14]. In contrast to systemic dpp4 inhibition by Linagliptin, hepatic *dpp4* knockdown in db/db mice did not significantly affect glucose metabolism. However, following siRNA treatment, we observed that hepatic steatosis was reduced and that hepatic mRNA expression of selected key genes was significantly lower compared to mice in control groups.

## Materials and methods

### Materials

All chemicals were purchased from Sigma-Aldrich (Munich, Germany), Merck (Darmstadt, Germany), or Roth (Karlsruhe, Germany). The reagents used for RNA isolation, cDNA preparation and qPCR were purchased from Thermo Fisher Scientific (Schwerte, Germany) unless stated otherwise.

### Cell culture and transfection of PC-3 cells

PC-3 cells were obtained from ATCC via LGC Standards (ATCC CRL_1435) and cultivated according ATCC’s instructions. Transfection of PC-3 cells with siRNAs was enhanced with Lipofectamine2000 (Invitrogen/Life Technologies, Karlsruhe, Germany) according to manufacturer’s instructions directly after seeding. The activity of a given *dpp4* siRNA was expressed as percent *dpp4* mRNA concentration in treated cells, relative to the *dpp4* mRNA concentration averaged across control wells.

### RNA isolation and quantitative real-time PCR

Total RNA was extracted from PC-3 cells and mouse livers using RNeasy Mini kit according to the manufacturer’s protocol. Animal tissues were immediately snap-frozen in liquid nitrogen after collection The target gene *dpp4* (TaqMan^®^ Gene Expression Assay: Mm00494549_m1) was measured by real time quantitative PCR. In addition, PCR using micro-fluidic cards in 96-well format was performed as described earlier [15].

### Animal studies

Female C57BL/6J (7 weeks old) as well as db/- and db/db mice (12 weeks old) were obtained from Charles River (Sulzfeld, Germany). Following a 2-week acclimatization period, mice were randomized into the respective treatment groups. During the experiment, mice had ad libitum access to filtered tap water and a standard rodent maintenance diet (Ssniff, Soest, Germany). Mice were group-housed at room temperature (20±2.0°C) in an environmentally controlled SPF-animal facility on a 12 h light-dark cycle. All experimental animal procedures were carried out at Sanofi Aventis Deutschland GmbH in Frankfurt, Germany in strict accordance with the terms of the German Animal Protection Law and international animal welfare legislation and rules. Sanofi as institution has an Institutional Animal Care and Use Committee (IACUC) termed AWB (Animal Welfare Body/ethics committee). The German authorities as well as the institutional AWB approved the presented studies including the use of animals.

**Fig 1:**
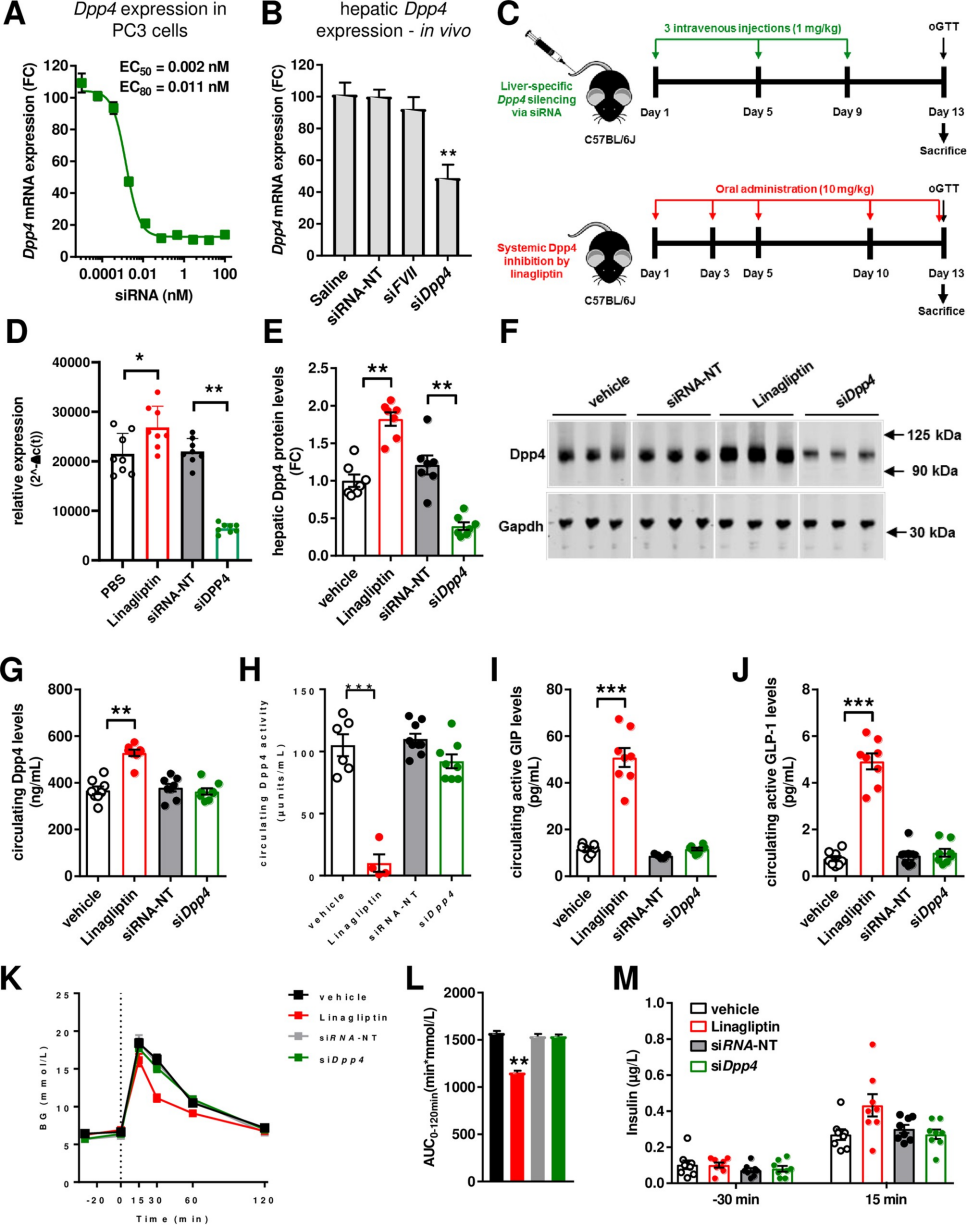
Liver-specific *dpp4* silencing does not improve glucose metabolism. (**A**) Dose-response curve upon transfection of PC3 cells with siRNA (n = 3). (**B**) Pilot mouse study to evaluate the siRNA efficacy. Wild-type C57BL/6J mice were injected three-times with *dpp4* siRNA (siDpp4, 1 mg/kg), negative control siRNA (luciferase; siRNA-NT) or factor VII siRNA (siFVII) and *dpp4* mRNA expression in the liver was analyzed by RT-PCR 7 days after treatment (n = 3; **p<0.005 vs. siRNA-NT (ANOVA, Tukey's multiple comparisons test). (**C**) Illustration of the mouse study design. Liver samples were taken after study termination (day 13) and *dpp4* mRNA expression (**D**) was analyzed by RT-PCR or protein abundance (**E**) by Western blot. (**F**) Representative Western Blot images show *dpp4* as well as Gapdh protein levels in liver samples. (**G**) Circulating *dpp4* protein levels as well as (**E**) circulating *dpp4* activity was measured three days after last dosing on day 13. (**F**) Quantification of active GLP-1 and (**G**) GIP at day 13. (**K**) Oral glucose tolerance test was performed after the 3rd injection, immediately before sacrifice. (**L**) Area under the curve during the oral glucose tolerance test. (**M**) Insulin levels before (-30 min) and after the oral glucose load (15 min). Data are mean values ± SEM, n = 6–8, **p<0.005 and ***p<0.0001 as indicated (ANOVA, Tukey's multiple comparisons test). FC = fold change.

**Fig 2:**
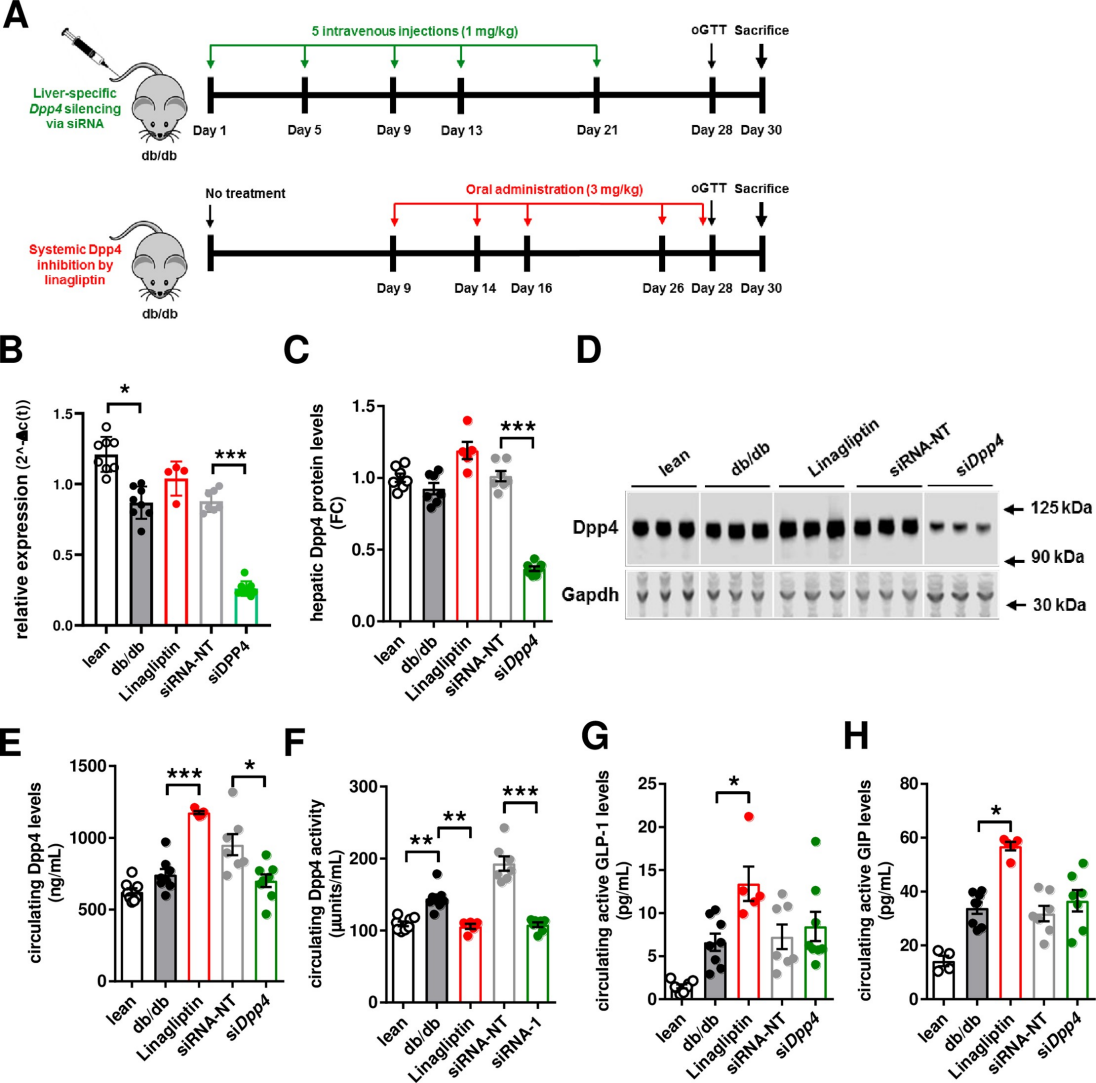
siRNA-mediated knock-down of hepatic *dpp4* in db/db mice. (**A**) Schematic illustration of the study design. (**B**, **C** and **D**) Liver samples were taken after study termination at day 30. (**B**) *dpp4* mRNA expression was analyzed by real-time PCR after indicated treatment. (**C**) Quantification of *dpp4* protein abundance. (**D**) Representative Western Blot images show *dpp4* protein levels after indicated treatment. (**E**) Circulating *dpp4* protein levels as well as (**F**) circulating *dpp4* activity was measured three days after last dosing at day 30. (**G**) Quantification of active GLP-1 and (**H**) GIP at day 30. Data are mean values ± SEM, n = 6–8, *p<0.05, **p<0.005 and ***p<0.0001 as indicated (ANOVA, Tukey's multiple comparisons test). FC = fold change.

Body weight was recorded twice weekly and at the day of sacrifice; food intake was analyzed once per week by weighing the food hoppers of mouse cages. The amount of consumed food per cage and week was then divided by the number of mice per cage and by the number of observation days to obtain an estimate of the average amount of food which each mouse consumed within 24 hours. C57BL/6J mice received 3 i.v. injections during 13 days, db/- and db/db 5 i.v. injections during 30 days (treatment schemes displayed in Figs 1C and 2A). Treatment groups received vehicle (PBS), the siRNA control or the *dpp4*-targeting siRNA (1 mg/kg). Liver-specific delivery of siRNAs was achieved using lipid nanoparticles (LNPs) based on the Axolabs' proprietary cationic lipid XL-10 technology (Axolabs, Kulmbach, Germany). This specific formulation is commercially available from Axolabs upon a custom-tailored client request (see www.axolabs.com for further details). A comparator group was treated by oral gavage once daily with the systemic *dpp4* inhibitor Linagliptin. On each treatment day, the oral treatment solution was prepared freshly by dissolving Linagliptin tablets in sterile DPBS (Thermo Fisher Scientific, Schwerte, Germany) to obtain a treatment solution containing 2 mg of Linagliptin per milliliter of DPBS. Mice were then dosed based on individual body weight to reach the respective target dose (3 mg/kg in db/db mice; 10 mg/kg in lean C57BL/6J mice).

For oral glucose tolerance tests (oGTT), mice were fasted for 6 h prior to the oral glucose load (1 g of glucose/kg body weight) and blood was sampled from the tail vein. In C57BL/6J mice, the last i.v. injection (3rd) was administered on day 9 of the experiment, oGTTs were performed on experimental day 13 and mice were sacrificed immediately thereafter. In db/- and db/db mice, the last i.v. injection (5th) was performed on day 21 of the experiment, oGTTs were performed on experimental day 28 and mice were sacrificed on experimental day 30. All mice were sacrificed under isoflurane anesthesia by cervical dislocation after a 6 h fasting period. Blood samples were collected, processed and stored as previously recommended for metabolic rodent studies [16, 17]. In brief, blood for EDTA-plasma was collected from the retrobulbar sinus in chilled Eppendorf tubes which were pre-filled with EDTA and a protease inhibitor cocktail including a specific *dpp4* inhibitor from Millipore (USA), a general protease-inhibitor from Sigma-Aldrich (USA) and an inhibitor of serine proteases (Roche Diagnostics; USA) to allow appropriate analysis of incretin hormones as previously described [16, 17].

### Western blotting

Mouse liver tissues (30mg) were lysed in a buffer containing 50 mM Tris/HCl (pH 7.4) and 1% sodium dodecylsulfate (SDS) supplemented with PhosSTOP and CompleteTM protease inhibitor cocktail (Roche Diagnostics; USA). After centrifugation for 30min at 4°C and 100,00 X g, protein concentration in the supernatant was determined using a BCA protein assay reagent kit (Thermo Fisher Scientific; Schwerte, Germany); the supernatants were then mixed with 4X Laemmli sample buffer containing denaturating SDS and heated for 20min to 70°C. Thereafter, 50 μg protein sample of the total cell lysate was separated by 4%-12% NuPAGE Bis-Tris Midi Gel electrophoresis using (Thermo Fisher Scientific; Schwerte, Germany) and transferred to a polyvinylidene fluoride membrane using a trans-Blot turbo transfer system (Bio-Rad, Feldkirchen, Germany). Membranes were blocked in a specific western blocking puffer (LI-COR Biosciences, Nebraska USA) and then incubated in an iBind Flex Western system (Invitrogen) with primary and secondary antibodies according to manufacturer’s instructions. The following primary antibodies used,: Mouse dpp4 (RnD systems, antibody clone AF954); gapdh (Update/Chemicom/Millipore, MAB374); atf-6 (Cell Signaling Technology, D4Z8V); phospho-PERK (Cell Signaling Technology, cat# 83416); phospho-eIF2α-Ser51 (Cell Signaling Technology, D9G8); eIF2α (Cell Signaling Technology, L57A5); and xpb-1s (Cell Signaling Technology, E9V3E). All antibodies were used at a 1/1000 dilution, except for anti-dpp4, which as diluted 1/2500. As secondary antibodies, species specific antibodies covalently linked to near-infra dyes (LI‐COR Biosciences, Lincoln, USA) were used. Imaging was performed on an Odyssey Fc Imaging System (LI‐COR Biosciences, Nebraska USA). Densitometric quantification of detected signals was performed using Image Studio version 5.0 software (LI‐COR Biosciences, Lincoln, USA).

### Biochemical analysis

Blood glucose from hemolyzed samples, serum triglycerides and serum cholesterol were determined using a Cobas 8000 system and the GLUC2, CHOL2 and TRIGL test kits from Roche Diagnostics (Mannheim, Germany), respectively. Mouse insulin and active GLP-1 were measured from EDTA plasma samples using chemiluminescent assays from Meso Scale Discovery (Gaithersburg, USA). GIP was measured using the Mouse active GIP ELISA kit from Crystal Chem Europe (Zaandam, The Netherlands), adiponectin was analyzed in plasma by a mouse adiponectin assay (MRP300) from R&D Systems (Minneapolis, M, USA). Circulating *dpp4* levels were determined using the Mouse *dpp4* ELISA kit from Abcam (Berlin, Germany). All assays were carried out according to the manufacturers’ instructions.

### Histopathology and image analysis

At study termination, liver tissue samples were preserved in 10% neutral buffered formalin, routinely processed for paraffin-embedding, sectioned at ~5 micron, and stained with hematoxylin and eosin (H&E) [14]. Histopathology slides were scanned at 20x magnification as digital whole slide images (WSI) using Aperio ScanScope XT2 digital slide scanner (Aperio Biosystems, Inc. Buffalo Grove, IL). Digital WSIs were evaluated for steatosis and inflammation by board-certified veterinary pathologists (SC, DB) using Aperio ImageScope viewer version 12.4.0 (Leica Biosystems). For quantitative assessment of lipid accumulation within hepatocytes, WSIs were subjected to automated image analysis using Visiopharm image analysis platform (VIS 2019.05; Hoersholm, Denmark). Briefly, the WSIs were first processed to delineate the hepatic parenchyma from blood vessels, bile ducts, sinusoids and other white space. Large and small diameter lipid droplets were then identified within hepatocytes based on their shape and size and enumerated as percent of total liver area. Total lipid area was obtained as a sum of the large droplet area and small droplet areas.

### Determination of *dpp4* activity

*Dpp4* activity in serum was measured with a fluorometric kit from Sigma Aldrich (Munich, Germany). 10 μl of serum was assayed as detailed by the manufacturer.

### Data and statistical analyses

All data are presented as mean ± SEM or standard deviation (SD) as indicated in the respective figure legends. GraphPad Prism 7 (La Jolla, USA) was used to calculate statistics and draw graphs. Statistical differences were calculated using one-tailed or two-tailed unpaired Student’s t-test with Welch’s correction or Mann-Whitney test depending on whether data passed normal distribution tests and two-way (repeated measurement) ANOVA adjusted for multiple testing and Tukey´s multiple comparisons test for post hoc analysis where appropriate. *P* < 0.05 was considered significant. ArrayStudio software (Version 10.0.1.118; Qiagen Omicsoft), was used to determine relative gene expression assessed by realtime PCR, either in single PCR reactions or in microfluidic cards.

## Results

### Design and in vitro characterization of *dpp4*-specific siRNAs

First, 24 siRNAs with a perfect match with mouse *dpp4* transcripts (NM_010074.3, NM_001159543.1) were designed and synthesized (S1 Table). 2'-O-methylation was used to provide sufficient siRNA stability and suppress siRNA-mediated stimulation of an immune response. Knock down efficacy was determined by screen in PC3 cells using two concentrations for each siRNA (S1 Fig panel A). Seven potent siRNAs were selected and IC_50_ measurements were performed (S1 Fig panels 1B-I). The siRNA with the lowest IC_50_ (XD-07132 alias siDpp4) was selected for additional characterization. All further experiments were performed with XC.07132 siRNA, which we cite as siDpp4 in subsequent figures.

### Liver-specific dpp4 silencing in lean and metabolic healthy C57BL/6J mice

To increase siRNA stability in bloodstream and improve cellular uptake by the liver we used a liposomal formulation which has been shown to result in a liver-specific knockdown of the siRNA target gene [14, 18]. In a first pilot study in mice, treatment with siDpp4 resulted in an effective reduction of *dpp4* mRNA in liver after three injections (1 mg/kg) by about 50%, whereas a negative control siRNA (luciferase; siRNA-NT) and another *dpp4*-unrelated siRNA (factor VII, siFVII), did not result in a *dpp4* knockdown in liver of mice (Fig 1B). Next, C57BL/6J mice were treated every fourth day with the targeting siDpp4 (1mg/kg), siRNA-NT as well as the oral *dpp4* inhibitor Linagliptin for a total of 13 days to elucidate the metabolic effects of liver-selective *dpp4* mRNA down-regulation (Fig 1C). After 13 days, body weight (in g; means±SEM; vehicle: 20.2±0.3, Linagliptin: 19.7±0.3, siRNA-NT: 19.9±0.2, siDPP4: 19.8±0.2), nor serum ALT (in U/L, means±SEM; vehicle: 41±4, Linagliptin: 39±3, siRNA-NT: 37±4, siDPP4: 43±3) and AST concentrations not were affected by treatment (in U/L, means±SEM; vehicle: 111±6, Linagliptin: 110±11, siRNA-NT: 114±14, siDPP4: 115±11). As expected, the hepatic expression of *dpp4* as well as *dpp4* protein levels were significantly reduced by ~75% after treatment (Fig 1D–1F). A single band at a molecular weight lower than 125kDa indicates that the siRNA knockdown affected presumably the monomeric form of the dpp4 protein (see also S6 Fig for a full size western). Reduced hepatic *dpp4* expression had no effect on circulating *dpp4* concentrations or on serum *dpp4* activity (Fig 1G and 1H). Similarly, plasma active GIP and GLP-1 concentrations were not altered after siRNA mediated *dpp4* silencing, too (Fig 1I and 1J). Daily treatment with Linagliptin resulted in increased hepatic *dpp4* protein expression as well as circulating *dpp4* levels, whereas *dpp4* activity was strongly inhibited (Fig 1D–1H). In accordance with reduced *dpp4* activity in plasma, circulating concentrations of active GIP and GLP-1 increased in Linagliptin treated mice compared to controls (Fig 1I and 1J). Analysis of oral glucose tolerance revealed improved glucose handling in Linagliptin treated mice, whereas hepatic *dpp4* silencing had no effect on blood glucose or insulin concentrations during oGTT in healthy mice (Fig 1K–1M).

### Liver-specific dpp4 knockdown in hyperglycemic db/db mice

To investigate the metabolic effects of liver-selective *dpp4* silencing in obese and diabetic animals, db/db mice were treated for four weeks with a total of five intravenous injections of the respective targeting or control siRNAs at a dose of 1 mg/kg (siDpp4 and siRNA-NT; Fig 2A). Hepatic *dpp4* mRNA expression as well as circulating *dpp4* activity was significantly increased in obese db/db mice compared to lean db/- control animals (Fig 2B and 2F). Treatment with siDpp4 resulted in clear reductions of hepatic *dpp4* mRNA and protein expression, by ~80% (mRNA) and ~65% (protein), respectively (Fig 2B–2D). Moreover, circulating *dpp4* concentrations and *dpp4* activity were reduced after siRNA-mediated silencing (Fig 2E and 2F). In contrast to mice treated chronically and daily with Linagliptin for four weeks, the reduction of circulating *dpp4* activity did not affect plasma concentrations of active GLP-1 or GIP (Fig 2G and 2H). Like in healthy C57BL/6 mice, treatment with Linagliptin in db/db mice increased circulating *dpp4* levels, while blocking *dpp4* activity (Fig 2E and 2F).

**Fig 3:**
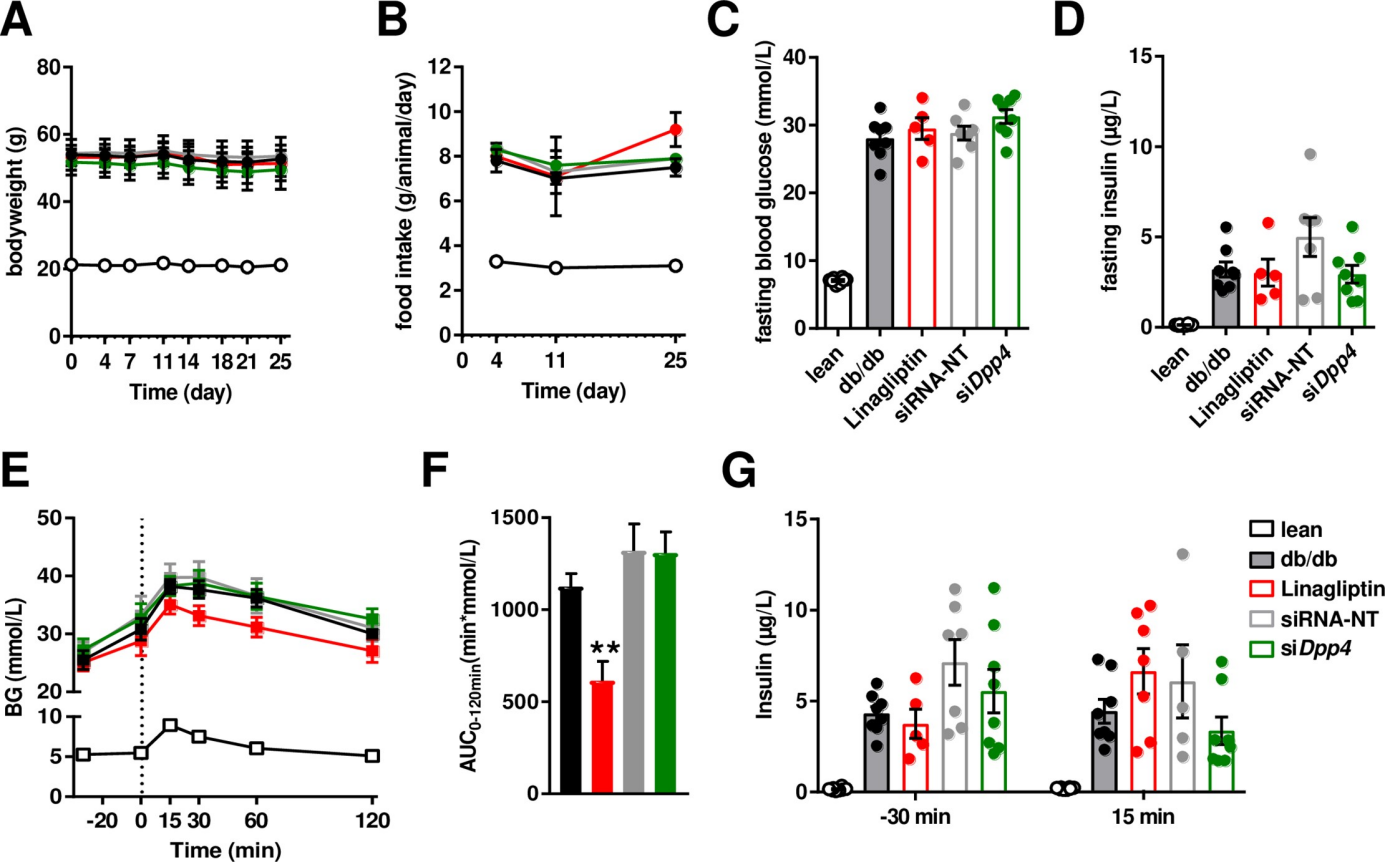
: Liver-specific *dpp4* silencing does not improve whole-body glucose metabolism. (**A**) Body weight and (**B**) food intake was measured during the study. (**C**) Fasting blood glucose and (**D**) fasting insulin was measured after study termination. (**E**) Oral glucose tolerance test was preformed 7 days after the 5th injection on day 28. (**F**) Baseline-corrected area under the curve during the oral glucose tolerance test. (**G**) Insulin concentrations before (-30 min) and after the oral glucose load (15 min).

The siRNA-mediated *dpp4* silencing for four weeks did not modulate the expression of other dipeptidyl peptidase (dpp) isoforms in liver. Isoforms such as *dpp7*, *dpp8* nor *dpp9*, all expressed in liver samples, were not affected (S2 Fig) by *dpp4* silencing. Silencing was also not associated with obvious adverse effects, such as changes in body weight (Fig 3A), food intake (Fig 3B) or circulating liver enzymes, i.e. ALT (in U/L, means±SEM; lean: 47±8, db/db: 140±17, Linagliptin: 120±1.3, siRNA-NT: 165±32, siDPP4: 144±7) or AST (in U/L, means±SEM; lean: 82±3, db/db: 111±6, Linagliptin: 114±10, siRNA-NT: 133±18, siDPP4: 138±12). As expected, circulating adiponectin concentrations were significantly higher in lean control mice compared to obese db/db mice. However, neither treatment with the targeting DPP4 siRNA nor Linagliptin significantly affected plasma adiponectin (Fig 3C right graph). This suggests that treatment had no effect on fat mass in db/db mice.

### Liver-specific dpp4 silencing does not improve whole-body glucose metabolism in db/db mice

To analyze how hepatic *dpp4* silencing impacts conditions of hyperglycemia and whole-body glucose metabolism, an oral glucose tolerance test (oGTT) was performed 4 weeks after start of treatment. In terminal samples, 6h fasting blood glucose as well as insulin concentrations were not significantly different between treatment groups (Fig 3C and 3D). As expected, oral glucose tolerance was improved in db/db mice treated chronically and daily with Linagliptin (Fig 3E and 3F). However, glucose tolerance was not different before and after glucose challenge in siDpp4 treated animals compared to controls (Fig 3E and 3F). Insulin concentrations during the oGTT were not different between the groups (Fig 3G).

### Effects of dpp4 silencing on hepatic lipid metabolism in db/db mice

**Fig 4:**
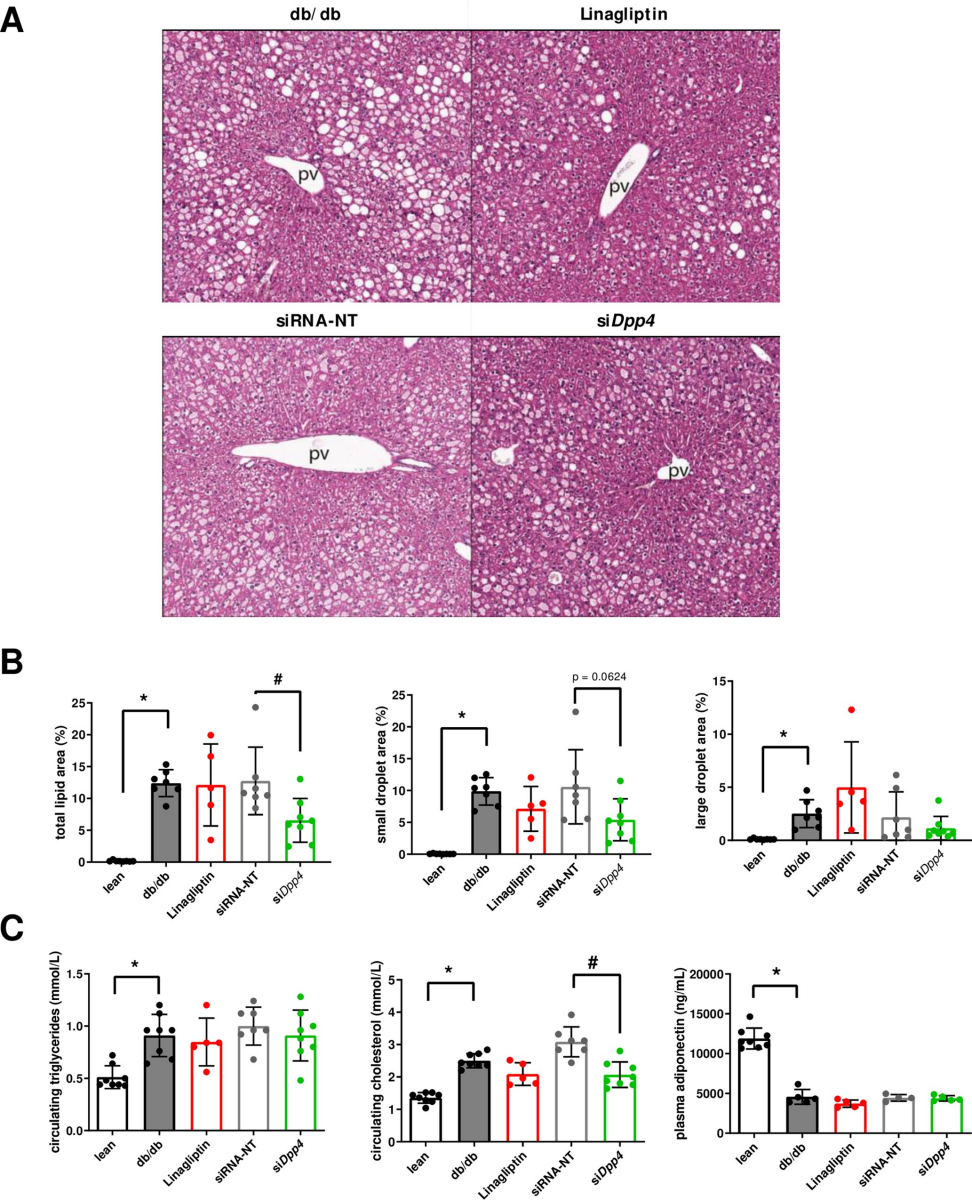
Effects of *dpp4* silencing on hepatic histology and lipid metabolism in db/db mice. (**A**) H&E images of liver depicting lipid accumulation (steatosis) in hepatocytes. All images at 100x magnification; pv = portal vein. (B) Quantitative analysis of the total lipid area, sub-analyzed for the small and large droplet area. (C) Circulating concentrations of triglycerides, cholesterol and adiponectin. Each solid circle represents liver sample from an individual mouse for each group (n = 5–8). Data are mean values ± standard deviation (SD), n = 5–8, * p<0.05.

Previous studies suggested that elevated hepatic *dpp4* expression resulted in hepatic steatosis, inflammation, liver damage and hypercholesterolemia. Therefore, we investigated the effects of hepatic *dpp4* silencing on plasma lipids and steatosis in liver. Histopathology of the liver revealed trends for reduced hepatic steatosis in siDpp4 and Linagliptin groups as compared to the db/db and siRNA-NT control groups (Fig 4A). Total percent liver lipid area was significantly reduced in the siDPP4 treated group as compared to the group treated with non-target siRNA (Fig 4B). Also the small lipid droplet area, indicative of microsteatosis in hepatocytes, appeared to be reduced following siDPP4 or Linagliptin treatment. However, this difference did not reach statistical significance. Concentration of circulating cholesterol, but not triglyceride was significantly reduced with siDpp4 treatment (Fig 4C).

### Expression changes following hepatic *dpp4* silencing

**Fig 5:**
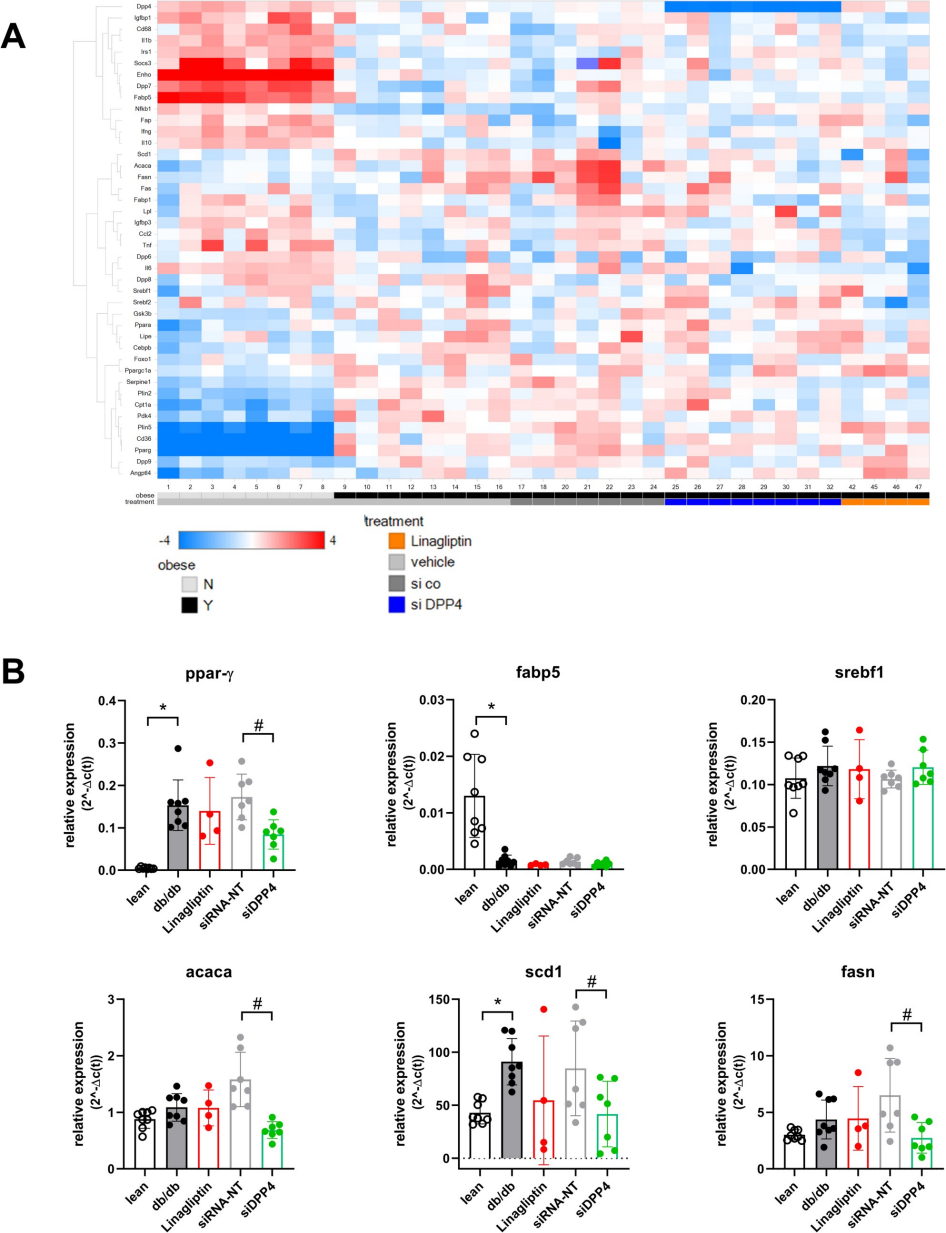
Hepatic gene expressions assessed by microfluidic card PCR. (A) Heatmap view showing a clustering of all assessed specific genes. A blue color indicates downregulation of the respective gene, whereas red square indicated up-regulation versus the mean of all samples. (B) Quantification view on selected single genes averaged for the treatment groups. Data are mean values ± SD, n = 5–8, *p<0.05 comparing obese db/db to lean db/- samples; ^#^p<0.05 comparing within the obese samples to the db/db control group (ANOVA, Tukey's multiple comparisons test).

We performed expression measurements on a selection of interesting genes by microfluidic card based PCR (Fig 5 and S2 Table). This selection included *dpp* isoforms and representative genes involved in lipid metabolism, inflammation and signaling. A heatmap view revealed clear differences between expression in lean db/- and obese db/db liver samples whereas more subtly nuances were observed in obese db/db samples from the different treatment groups (Fig 5A). Three genes direclty involved in fatty acid synthesis, namely acetyl-CoA carboxylase 1 (*acaca*), stearoyl-CoA desaturase (*scd1*), fatty acid synthase (*fasn*) were reduced by hepatic *dpp4* silencing (Fig 5B). Proliferator activated receptor gamma (*pparg*) was highly upregulated in obese conditions; it was reduced after chronic siDpp4 treatment only. Treatment with Linagliptin treatment did not modulate the expression of these four genes.

The expression of sterol regulatory element binding transcription factor 1 (*srebf1*) was neither modulated on mRNA level (Fig 5) nor on protein level assessed by western blotting (S3 Fig). Expression of further genes like insulin receptor substrate 1 (*irs1*), forkhead box protein O1 (f*oxo1*) as well as glycogen synthase kinase 3 beta (*gsk3b*) was also neither affected by siDpp4 nor by Linagliptin treatment (S2 Table).

### Assessment of hepatic inflammation

Several inflammatory markers were downregulated in obese db/db versus lean db/- liver samples; among them were interleukin-6 (IL-6), interleukin-10 (IL-10), and chemokine (C-C motif) ligand 2 (*Ccl-2*), tumor necrosis factor alpha (*TNFα*), interleukin-beta (*IL1β*) and interferon-gamma (Infg) as depicted in S4 Fig. Furthermore, plasminogen activator inhibitor-1 (serpine1) was upregulated in obese db/db versus lean db/- mice. Both siDPPA and Linagliptin reduced serpine-1 expression close to levels in lean db/- mice. Chronic Linagliptin treatment reduced the hepatic mRNA expression and plasma protein levels of *Il-6* (55% reduction). Hepatic specific knockdown of *dpp4* also reduced circulating IL-6 to the similar extent, however, the reduction was statistically not significant (*p* = 0.06).

On a histological level, hepatic inflammation was not a prominent feature of the db/db mouse model in our study (see Fig 4A). Minimal inflammatory foci observed in only a few mice from all study groups including the lean controls were interpreted to be at acceptable limit for these mouse strains. Although these observations do not corroborate lower plasma Il-6 concentrations following Linagliptin or siDpp4 treatment in db/db mice, a potential treatment effect on Il-6 producing cells in other tissues besides the liver cannot be excluded.

### Assessment of hepatic mitochondrial protein response

A signaling network, originating from mitochondria and described as unfolded protein response (UPR), has recently been described in the context of liver disease conditions [19]. We measured activation of several factors in this network by western blotting as outlined by Oslowski and Urano [20]. No activation or modulation could be observed for several factors like atf-6, phospho-perk, phospho-eIF2α and xbp-1 (S5 Fig).

## Discussion

Our data demonstrate that the reduction of *dpp4* exclusively in the liver is not sufficient to improve glucose metabolism in obese and hyperglycemic mice. Despite the knock-down of hepatic *dpp4* in chronically siRNA-treated mice, no effects on plasma active incretin hormone concentrations were observed. However, decreased hepatic *dpp4* expression was associated with a reduced expression of genes involved in lipid metabolism and reduced signs of hepatic steatosis and finally resulted in a reduction of circulating total cholesterol concentrations. Systemic inhibition of the enzymatic *dpp4* activity by Linagliptin resulted in a different outcome and mice displayed significantly improved glucose handling.

Recent publications have shown that obesity promotes liver *dpp4* production, and liver-specific *dpp4* transgenic mice displayed an elevated systemic *dpp4* activity and diminished GLP-1 levels [9–12, 21]. These studies have been performed in mice with diet induced obesity (DIO), but without apparent hyperglycemia. In contrast to the typically absent or mild hyperglycemia in DIO mice, db/db mice display obesity and strong hyperglycemia simultaneously. In such a background, we also observed an increased hepatic *dpp4* mRNA expression as well as elevated circulating *dpp4* activity like in DIO mice in earlier studies. In our study, the siRNA mediated hepatic silencing of *dpp4* resulted in a reduction of a broad protein band with a molecular weight of about 110kDa after separation by denaturing electrophoresis, presumably the dpp4 monomer. Using different, less denaturing conditions, Wang et al detected a second band at 160kDa and assigned it as dpp4 dimer [22]. As demonstrated in our experiments, the decreased circulating *dpp4* protein concentrations after siRNA knockdown are in concert with lower *dpp4* activity in plasma. However, the reduction in plasma *dpp4* activity did not affect incretin cleavage or glucose tolerance. In accordance with our data in db/db mice, Ghorpade and colleagues observed in DIO mice no changes in plasma active GLP-1 or GIP concentrations after silencing of hepatocyte *dpp4* in DIO mice, although they observed a reduced plasma *dpp4* activity that was comparable to sitagliptin [12]. Furthermore, Varin et al. observed, again in DIO mice, reduced circulating *dpp4* protein levels as well as lower *dpp4* activity after a liver specific-*dpp4* knockdown. In agreement with our study in db/db mice, DIO mice with the liver specific DPP4 knockdown displayed unchanged glucose tolerance and no significant effect on plasma levels of active GLP-1 and GIP compared to controls [21]. Collectively, our data and that found in literature confirm that liver-derived *dpp4* contributes to systemic *dpp4* concentrations in several mouse models. The finding that hepatocyte-secreted *dpp4* is dispensable for incretin cleavage and glucose control in diabetic mice may be extended to conditions of obesity and hyperglycemia.

Our findings related to systemic glucose control are in conflict to published data from Ghorpade et al., who showed that silencing of hepatic *dpp4* substantially improves glucose tolerance in obese and insulin-resistant DIO mice [12]. These authors postulated that a major mechanism behind improved glucose metabolism consists of the reduction of inflammation in visceral adipose tissue [12]. Those findings and conclusions were challenged by Varin et al. showing that knockdown of hepatocyte-derived *dpp4* reduced liver cytokine expression, and partially attenuated inflammation in adipose tissue. In contrast to Ghorpade et al., but in line with our data, Varin et al., did not observe significant changes in glucose homeostasis in these mice [21]. Two limitations of our studies are worth noting in this context. First, the siRNA nanoparticle technology applied in our study still resulted in a residual hepatic *dpp4* expression of ~25%, that could be still too high in order to induce pronounced effects on glucose metabolism. Second, we focused our studies on liver and did not investigate in detail adipose tissue interference beyond measuring circulating adiponectin concentrations. Unchanged adiponectin concentrations with siRNA treatment indicate no strong effect on adipose tissue mass.

In obese DIO mice, it is well documented that systemic *dpp4* inhibitors exert anti-inflammatory properties [23, 24]. In our study with obese and hyperglycemic db/db mice, we were able to confirm that systemic dpp4 inhibition has anti-inflammatory effects. In addition to a potential role of dpp4 in inflammatory processes, Wang and co-workers could demonstrate in a tetrachloride-induced model of liver injury that dpp4 has an important pro-fibrotic role, presumably through its enzymatic activity affecting metabolic and inflammatory mechanisms [22]. In our study, we could not further investigate this finding as liver fibrosis was not overtly present in our db/db mouse cohort. Linagliptin treatment led to a reduction of cytokine expression in the liver and lowered circulating IL-6 levels in db/db mice. Moreover, treatment with the siRNA targeting *dpp4* resulted in lower hepatic *Il6* mRNA expression and showed a trend on reducing circulating IL-6 levels (*p* = 0.06). Therefore, dpp4 synthesized in the liver may contribute to anti-inflammatory efficacy. This hypothesis is also indirectly supported by previous experiments showing that silencing of *dpp4* in primary human adipocytes has no effect on inflammation, as measured by MCP1 and IL-6 production [25]. Despite those similarities to other studies, we collected evidence that within the livers of db/db mice, tissue inflammation does not play a major role, e.g. in explaining a reduction of steatosis. On a histological level, minimal inflammatory foci could be observed in obese and hyperglycemic db/db liver tissues not different to lean and normoglycemic db/- controls. Furthermore, several inflammatory markers were down- and not upregulated when assessed by gene expression.

We also investigated the impact of liver-specific *dpp4* silencing on hepatic lipid metabolism. It has previously been shown that liver *dpp4* expression is elevated in humans with ectopic fat accumulation in the liver and that hepatic *dpp4* overexpression in mice is accompanied with liver steatosis, liver damage and hypercholesterolemia [10, 11]. In the current study, histological evaluation of liver sections revealed that db/db mice, treated with siRNA targeting *dpp4*, displayed a reduced liver lipid area when compared to the siRNA control group. Treatment with Linagliptin failed to reduce liver lipids to a similar level in our study which is in contrast to earlier publications, showing that Linagliptin treatment alleviates hepatic steatosis and inflammation in mouse models of NASH [26–28]. In another study, Kern *et al*. also treated db/db mice with Linagliptin (3 mg/kg). After 8 weeks of daily treatment, the investigators observed a moderate reduction in liver lipid content compared to mice treated with vehicle only [29]. This finding suggests that daily treatment for a longer period than four weeks is required in order to detect differences in liver lipid content with Linagliptin treatment in db/db mice. Furthermore, it should be noted that in our study, db/db mice were fed with a standard diet that did not specifically induce NASH. The lack of marked fibrosis and inflammation in our mouse model could potentially explain the discrepant findings compared to previous studies investigating the treatment effect of Linagliptin in dedicated rodent NASH models.

In *dpp4* siRNA treated mice, we observed reduced circulating cholesterol concentrations as well as increased hepatic expression of genes involved in lipid metabolism, namely *pparg acaca*, *scd1*, *fasn*. The latter three genes code for important enzymes in de-novo synthesis of fatty acids and lipids and hence their reduction directly explains decreased levels of hepatosteatosis. We also measured the expression of transcription factors like sterol regulatory element binding transcription factor 1 (*srebf1*), a known regulator of *acaca*, *scd1*, and *fasn* and a key mediator in liver steatosis [30]. However, no regulation of *srebf1* could be observed neither between db/- and db/db mice nor by treatment with Linagliptin or siDPP4. An alternative transcription factor may be *pparg*. Chronic treatment of specific apo-lipoprotein-B modified mice with antisense oligonucleotides targeting *pparg* resulted in strong reductions of liver expression of *fasn* and *acaca*. This seems to be one of the few known evidences for such an upstream regulation [31]. The precise mechanism how liver *dpp4* is linked to *pparg* repression remains currently unknown and needs to be addressed in further, more unbiased measurements, e.g. by RNA-sequencing. We also observed that the inhibition of the enzymatic *dpp4* activity by Linagliptin did not result in similar effects as observed with the siRNA mediated *dpp4* reduction pointing to a direct effect of hepatocyte-derived *dpp4* on liver lipid metabolism independent of the enzymatic activity. This is supported by studies showing that *dpp4* can directly bind to cell-surface receptors to induce an intracellular signaling cascade [12, 32–34]. With Linagliptin, upregulation of hepatic *dpp4* mRNA and protein or circulating protein could be observed consistently, e.g. in healthy C57BL/6 mice. This is in accordance with similar results observed by Varin et al. in obese DIO mice [9] who proposed an auto-regulatory feedback loop linking reduction of enzymatic *dpp4* activity to augmentation of *dpp4* production. Further studies are needed to confirm the role of hepatic *dpp4* independent of its enzymatic activity.

In summary, the liver specific silencing of *dpp4* expression by siRNA affects metabolism in obese and hyperglycemic db/db mice differently when compared to systemic inhibition with an established *dpp4* inhibitor. In our study, no improvement of impaired glucose tolerance was observed with siRNA treatment in line with some but not all previously published studies in obese DIO mice using different means to reduce hepatic *dpp4* expression. However, treatment with the siRNA targeting dpp4 resulted in a reduction of liver steatosis and expression of key genes in lipid metabolism. Liver specific targeting of this pathway may specifically improve liver disease conditions without modulation of systemic glucose tolerance.

## Supporting information

S1 Fig *in vitro* characterization of *dpp4*-specific siRNAs. (**A**) PC3 cells were transfected with 50 or 100nM siRNA and expression of *dpp4* mRNA was analyzed by RT-PCR. Results are presented as fold-change relative to controls. (**B**-**H**) IC_50_ measurements were performed in PC3 cells. **(I)** Summary table of EC_50_ as well as EC_80_ values obtained from dose-response curves. FC = fold change
(TIF)

S2 Fig Liver expression of *dpp* isoforms in db/- and db/db mice. Expressions were assessed in the course of the microfluidic card PCR. Results are presented as relative expressions. Data are mean values ± SD, n = 5–8, *p<0.05 comparing obese db/db to lean db/- samples; ^#^p<0.05 comparing within the obese samples to the db/db control group (ANOVA, Tukey's multiple comparisons test).
(TIF)

S3 Fig Expression of srebf1 assessed by western blotting in liver samples of db/- and db/db mice. Samples were subjected to gel electrophoresis and blotted on PVDF membranes, before incubation with specific primary antibodies listed in material and methods of the main manuscript body. Images were scanned using a Licor Oddyssee-Fc system. Whereas gapdh as loading control could be clearly visualized, no band for srebf1 could be detected (expected m.wt. = 50kDa)
(TIF)

S4 Fig Liver expression of inflammation markers and plasma concentrations of IL-6 and Ccl-2 in db/- and db/db mice. Expressions were assessed in the course of the microfluidic card PCR. Plasma IL-6 and Ccl-2 were determined by specific immunoassays. Results are presented as relative expressions and total plasma concentrations. Data are mean values ± SD, n = 5–8, *p<0.05 comparing obese db/db to lean db/- samples; ^#^p<0.05 comparing within the obese samples to the db/db control group (ANOVA, Tukey's multiple comparisons test).
(TIF)

S5 Fig Unfolded protein response assessed by western blotting in liver samples of db/- and db/db mice. Samples were subjected to gel electrophoresis and blotted on PVDF membranes, before incubation with specific primary antibodies listed in material and methods of the main manuscript body. As control, differentiated mouse skeletal muscle cells C2C12 were treated without (-) and with (+) thapsigargin (1μM, 20min). Images were scanned using a Licor Oddyssee-Fc system.
(TIF)

S6 Fig Full-Size western blot against dpp4 in liver samples of db/- and db/db mice. Samples were subjected to denaturing SDS gel electrophoresis. After blotting and blocking, membranes were incubated with an anti-dpp4 antibody (RnD Systems, AF954, 1/2500 dilution). In accordance with manufacturer’s information, the antibody detects a single band with a slightly lower molecular weight than a 125 kDa molecular weight marker, presumably the monomeric dpp4 subunit. The intensity of staining of this single band is strongly reduced in liver samples of mice treated with siRNA against dpp4 (siDpp4).
(TIF)

S1 Table Target mRNA sequence of each *dpp4* siRNA duplex. Capital letters: RNA; small letters: 2’-Ome; dT: DNA-T; s: Phosphorothioate
(PDF)

S2 Table Excel file with data obtained by microfluidic card PCR of db/db and db/- liver samples. (XLSX)
