# Supplementary figures and images for: A siRNA mediated hepatic dpp4 knockdown affects lipid, but not glucose metabolism in diabetic mice
Source: PLoS One. 2019 Dec 3;14(12):e0225835. doi: 10.1371/journal.pone.0225835 (PMC6890245; doi:10.1371/journal.pone.0225835)

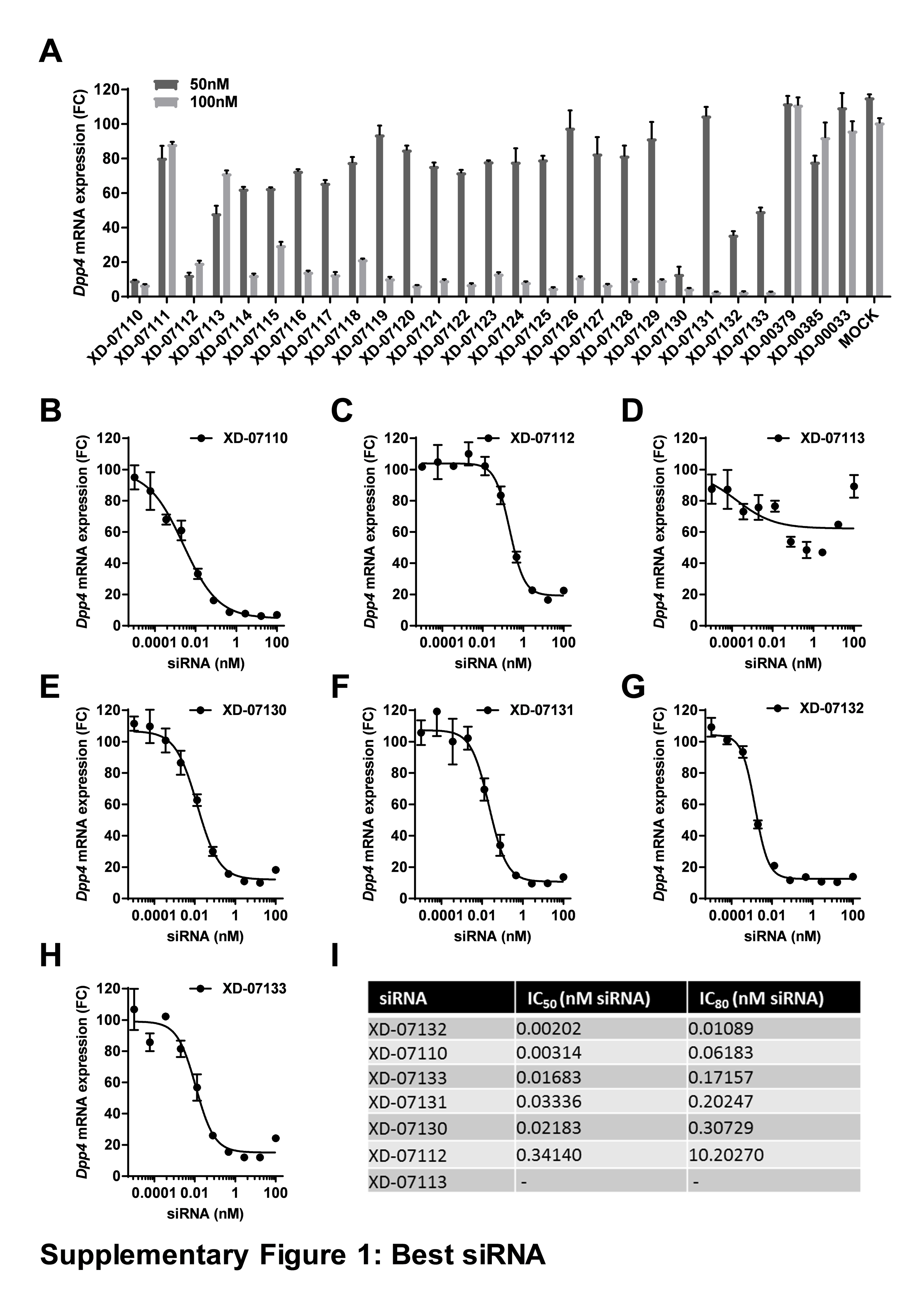

Supplement: S1 Fig — (A) PC3 cells were transfected with 50 or 100nM siRNA and expression of dpp4 mRNA was analyzed by RT-PCR. Results are presented as fold-change relative to controls. (B-H) IC50 measurements were performed in PC3 cells. (I) Summary table of EC50 as well as EC80 values obtained from dose-response curves. FC = fold change (TIF) [file pone.0225835.s001.tif]

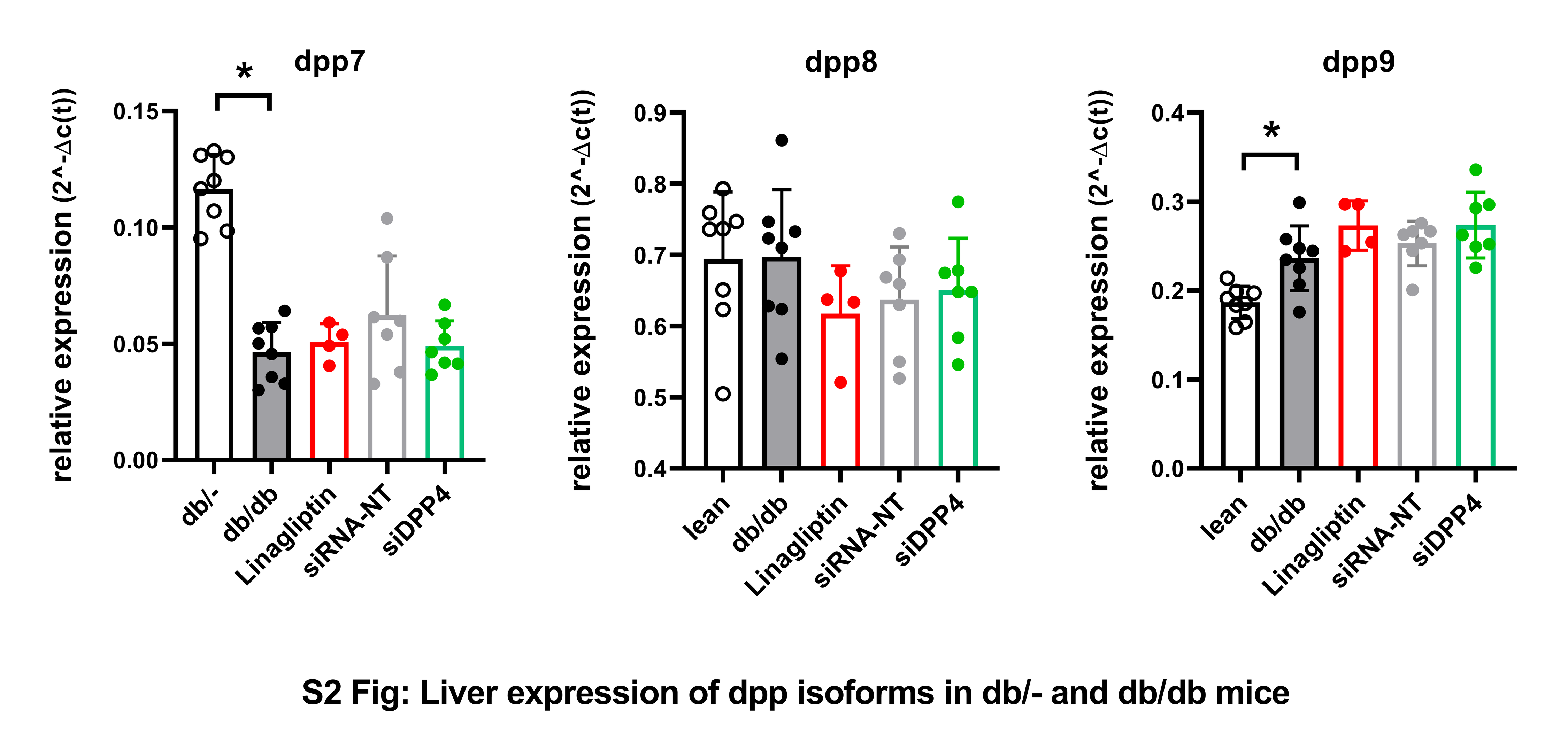

Supplement: S2 Fig — Expressions were assessed in the course of the microfluidic card PCR. Results are presented as relative expressions. Data are mean values ± SD, n = 5–8, *p<0.05 comparing obese db/db to lean db/- samples; #p<0.05 comparing within the obese samples to the db/db control group (ANOVA, Tukey's multiple comparisons test). (TIF) [file pone.0225835.s002.tif]

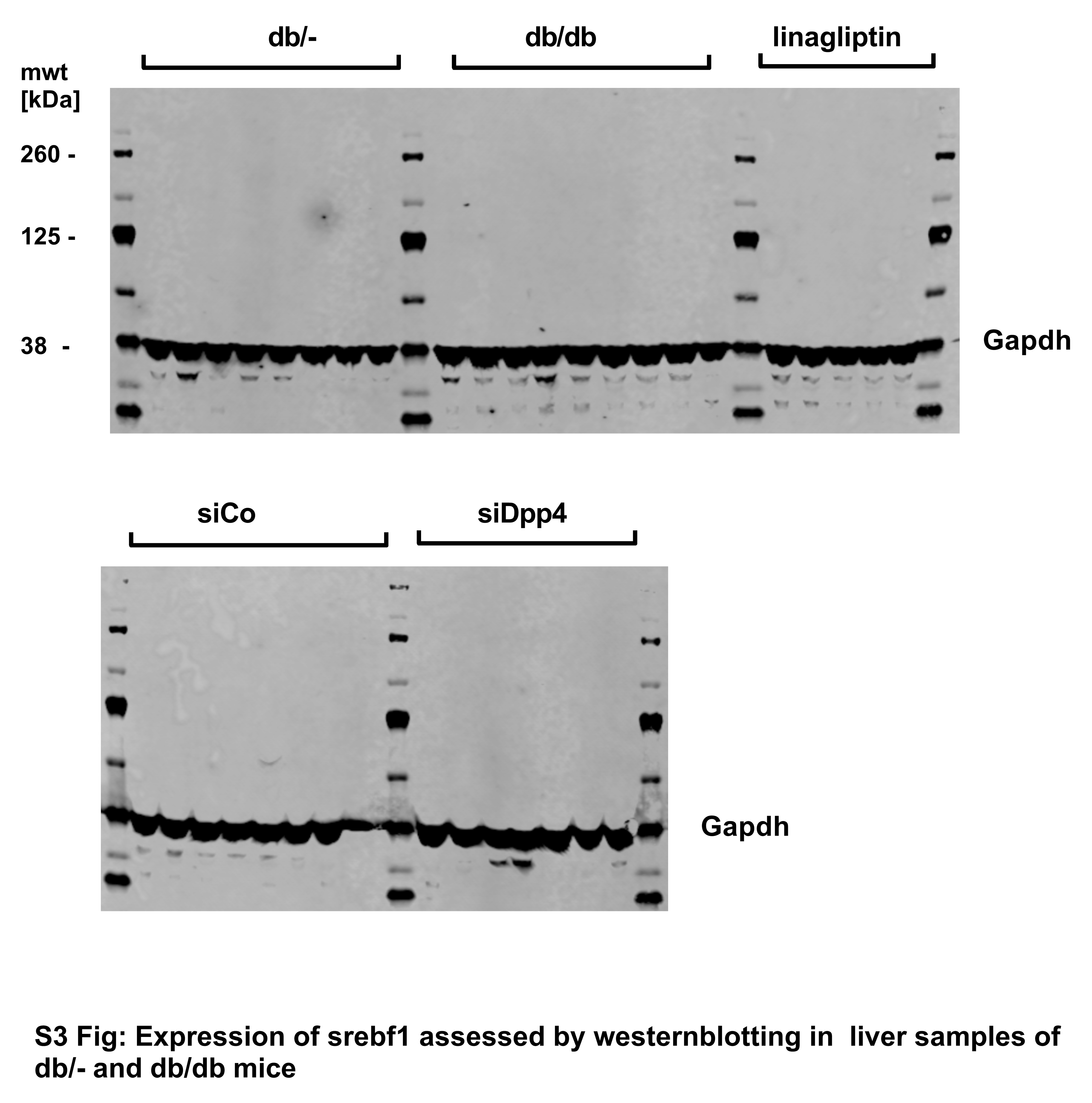

Supplement: S3 Fig — Samples were subjected to gel electrophoresis and blotted on PVDF membranes, before incubation with specific primary antibodies listed in material and methods of the main manuscript body. Images were scanned using a Licor Oddyssee-Fc system. Whereas gapdh as loading control could be clearly visualized, no band for srebf1 could be detected (expected m.wt. = 50kDa) (TIF) [file pone.0225835.s003.tif]

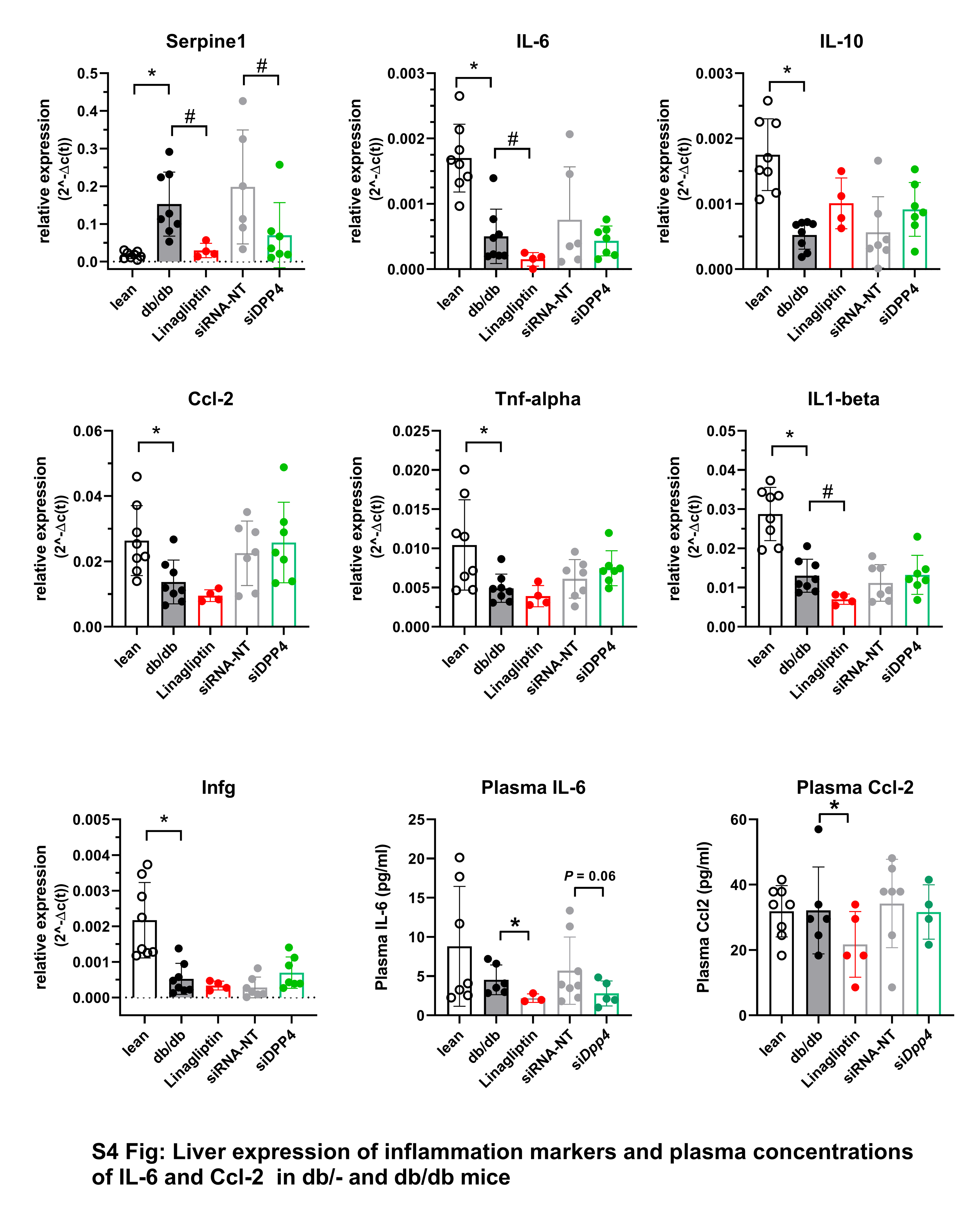

Supplement: S4 Fig — Expressions were assessed in the course of the microfluidic card PCR. Plasma IL-6 and Ccl-2 were determined by specific immunoassays. Results are presented as relative expressions and total plasma concentrations. Data are mean values ± SD, n = 5–8, *p<0.05 comparing obese db/db to lean db/- samples; #p<0.05 comparing within the obese samples to the db/db control group (ANOVA, Tukey's multiple comparisons test). (TIF) [file pone.0225835.s004.tif]

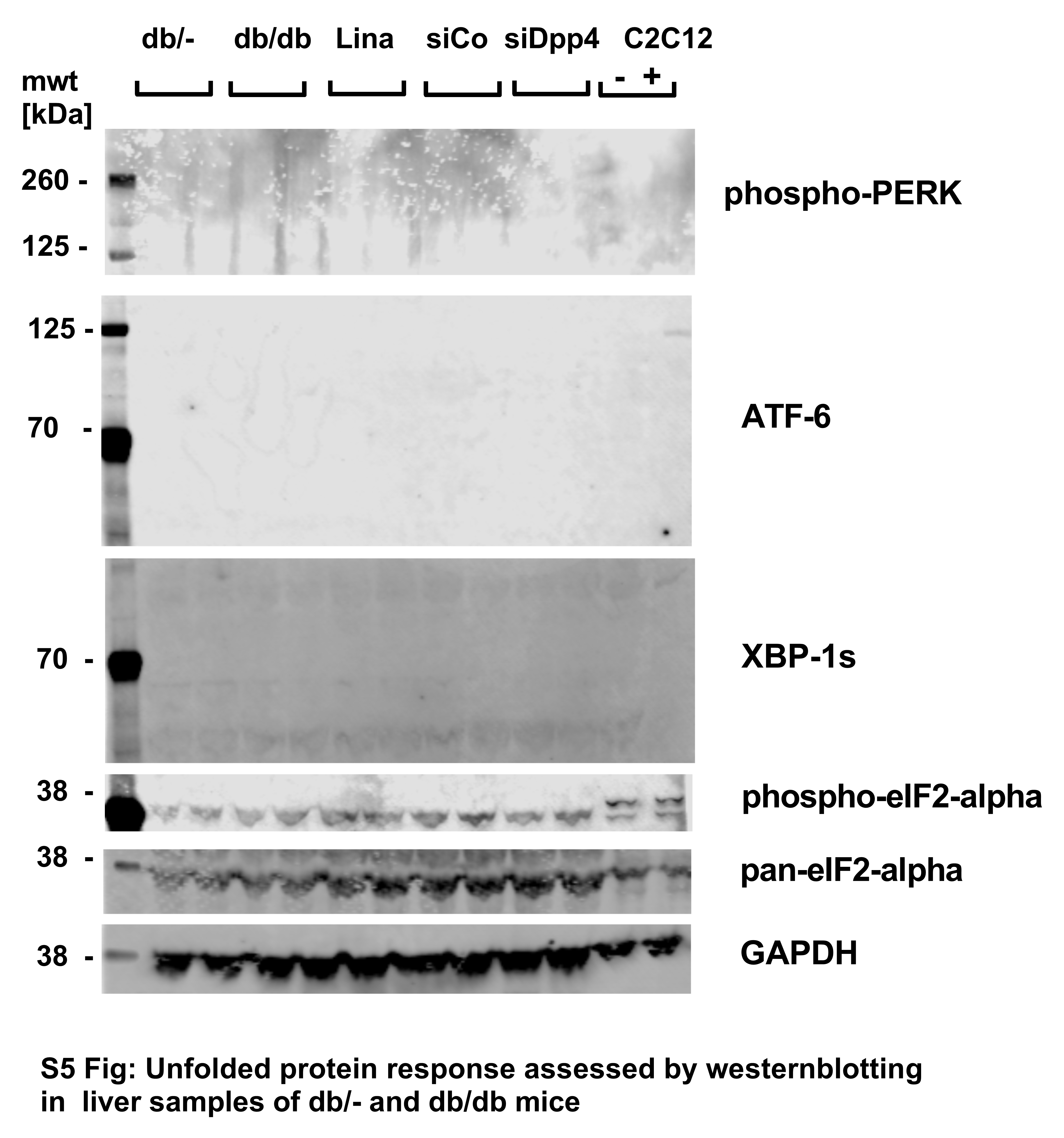

Supplement: S5 Fig — Samples were subjected to gel electrophoresis and blotted on PVDF membranes, before incubation with specific primary antibodies listed in material and methods of the main manuscript body. As control, differentiated mouse skeletal muscle cells C2C12 were treated without (-) and with (+) thapsigargin (1μM, 20min). Images were scanned using a Licor Oddyssee-Fc system. (TIF) [file pone.0225835.s005.tif]

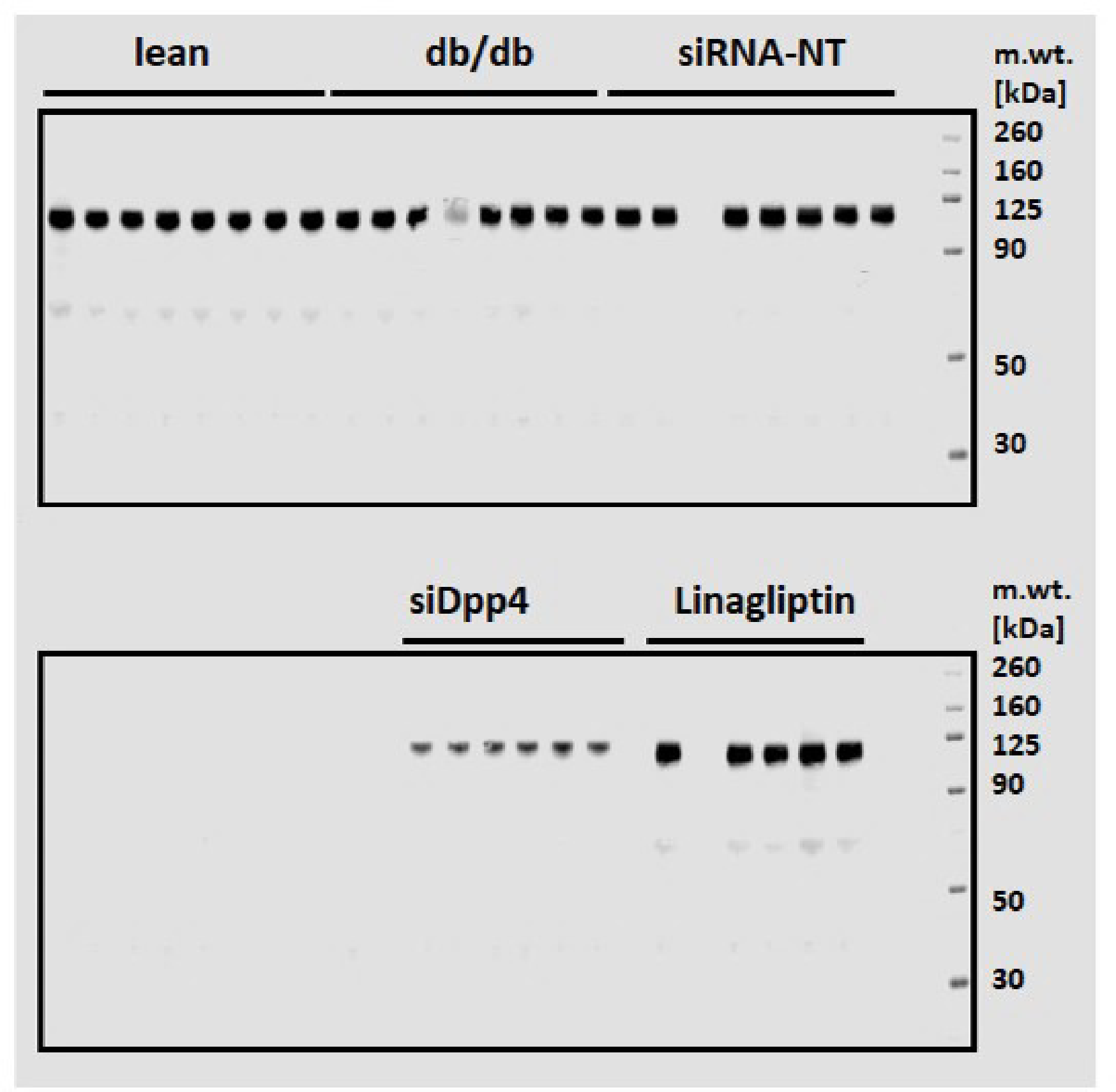

Supplement: S6 Fig — Samples were subjected to denaturing SDS gel electrophoresis. After blotting and blocking, membranes were incubated with an anti-dpp4 antibody (RnD Systems, AF954, 1/2500 dilution). In accordance with manufacturer’s information, the antibody detects a single band with a slightly lower molecular weight than a 125 kDa molecular weight marker, presumably the monomeric dpp4 subunit. The intensity of staining of this single band is strongly reduced in liver samples of mice treated with siRNA against dpp4 (siDpp4). (TIF) [file pone.0225835.s006.tif]
