# Supplementary material for: A siRNA mediated hepatic dpp4 knockdown affects lipid, but not glucose metabolism in diabetic mice
Source: PLoS One. 2019 Dec 3;14(12):e0225835. doi: 10.1371/journal.pone.0225835 (PMC6890245; doi:10.1371/journal.pone.0225835)
Supplement: S1 Table — Capital letters: RNA; small letters: 2’-Ome; dT: DNA-T; s: Phosphorothioate (PDF) [file pone.0225835.s007.pdf]

ID	sense strand sequence (5´-3´)	antisense strand sequence (5´-3´)
XD-07110	caccGuGGAAGGuucuucudTsdT	AGAAGAACCUUCcACGGUGdTsdT
XD-07111	gcuGcGcuuGucAccAucAdTsdT	UGAUGGUGAcAAGCGcAGCdTsdT
XD-07112	gaAuAcAAcuAcGuGAAGcdTsdT	GCUUcACGuAGUUGuAUUCdTsdT
XD-07113	aacuAcGuGAAGcaAuGGAdTsdT	UCcAUUGCUUcACGuAGUUdTsdT
XD-07114	ggAAuAAcuGAcugGGuuudTsdT	AAACCcAGUcAGUuAUUCCdTsdT
XD-07115	aacuGAcuGGGuuuAuGAAdTsdT	UUcAuAAACCcAGUcAGUUdTsdT
XD-07116	cauuGAAuAcuccuucuAudTsdT	AuAGAAGGAGuAUUcAAUGdTsdT
XD-07117	uuAucuAuAcuAcauuAGudTsdT	ACuAAUGuAGuAuAGAuAAdTsdT
XD-07118	aaAuccAAGAAAuauccucdTsdT	GAGGAuAUUUCUUGGAUUUdTsdT
XD-07119	agAAAuAuccucuacuAuudTsdT	AAuAGuAGAGGAuAUUUCUdTsdT
XD-07120	aauAuccucuAcuauuAGAdTsdT	UCuAAuAGuAGAGGAuAUUdTsdT
XD-07121	cauAGuAGcuAGcuuuGAcdTsdT	GUcAAAGCuAGCuACuAUGdTsdT
XD-07122	auAAGAucAuGcAuGcAAudTsdT	AUUGcAUGcAUGAUCUuAUdTsdT
XD-07123	agAucAuGcAuGcaAucAAdTsdT	UUGAUUGcAUGcAUGAUCUdTsdT
XD-07124	auGcAuGcAAucAacAGAAdTsdT	UUCUGUUGAUUGcAUGcAUdTsdT
XD-07125	gcAuGcAAucAAcaGAAGAdTsdT	UCUUCUGUUGAUUGcAUGCdTsdT
XD-07126	gguccuGGGAucGgGAAGudTsdT	ACUUCCCGAUCCcAGGACCdTsdT
XD-07127	gaucGGGAAGuGGcGuGuudTsdT	AAcACGCcACUUCCCGAUCdTsdT
XD-07128	ggAAGuGGcGuGuucAAGudTsdT	ACUUGAAcACGCcACUUCCdTsdT
XD-07129	uacuAuGAcucAGuGuAcAdTsdT	UGuAcACUGAGUcAuAGuAdTsdT
XD-07130	gaAGAcAAccuuGaccAuudTsdT	AAUGGUcAAGGUUGUCUUCdTsdT
XD-07131	uuAAAcAAGuuGAguAccudTsdT	AGGuACUcAACUUGUUuAAdTsdT
XD-07132	aguuGAGuAccuccuuAuudTsdT	AAuAAGGAGGuACUcAACUdTsdT
XD-07133	guAccuccuuAuucAuGGAdTsdT	UCcAUGAAuAAGGAGGuACdTsdT

Table 1: Target mRNA sequence of each Dpp4 siRNA duplex. Capital letters: RNA; small letters: 2'-Ome; dT: DNA-T; s: Phosphorothioate

S1 Table: Target mRNA sequence of each dpp4 siRNA duplex
